# The intervention effect of caffeine oral tablets on mental fatigue induced by nap deprivation: based on assessments of heart rate variability, cognitive performance and subjective fatigue—a pilot study

**DOI:** 10.3389/fpubh.2026.1830975

**Published:** 2026-06-18

**Authors:** Yong-Zheng Fan, Shuai-Yi Li, Xiao-Lei Lin, An-Na Zhang, Yu Wang, Ke-Feng Huang, Wen-Xian Song

**Affiliations:** The 991st Hospital of Joint Logistic Support Force of People's Liberation Army, Xiangyang, Hubei, China

**Keywords:** heart rate variability, low-dose caffeine oral tablets, mental fatigue, nap deprivation, pilot study

## Abstract

**Background:**

This study aimed to investigate the feasibility of inducing mental fatigue (MF) through nap deprivation, based on monitoring heart rate variability (HRV), cognitive performance, and subjective fatigue levels. Additionally, the intervention effect of caffeine oral tablets (COT) on MF was evaluated.

**Methods:**

This was an open-label, non-randomized, single-sequence crossover pilot study. The caffeine content in COT was quantified using high-performance liquid chromatography (HPLC). Ten healthy volunteers who habitually took naps were enrolled. Participants underwent nap deprivation to induce MF. Various parameters were collected at different time points, including the Karolinska Sleepiness Scale (KSS), Attention Network Task (ANT), and HRV metrics. Changes in each parameter before and after COT intervention were analyzed.

**Results:**

Following nap deprivation, there was a significant increase in KSS scores for the control group compared to baseline measurements taken after 2 h of sleep deprivation (*P* < 0.01). No significant changes were observed in ANT performance; however, HRV exhibited variations influenced by circadian rhythms—showing increased volatility in time-domain parameters and differing trends in frequency-domain parameters without statistical significance. After administration of low-dose COT to experimental participants, KSS scores did not significantly rise; conversely, correct response rates (CR) on ANT improved significantly (*P* < 0.05), alongside notable changes in both time-domain and frequency-domain HRV parameters (*P* < 0.05).

**Conclusions:**

In this pilot study, the alignment between subjective and objective indices suggests that low-dose COT is associated with favorable changes in the MF state of volunteers following sleep deprivation. However, due to the small sample size and open-label design, these findings should be considered preliminary and warrant confirmation in larger, randomized controlled trials.

## Introduction

Mental fatigue (MF) is a gradual and cumulative process characterized by feelings of boredom toward tasks, diminished motivation to exert effort, fatigue, and inhibition of cognitive activities. This phenomenon can lead to decreased work efficiency and alertness (1, 2). In severe instances, it may even result in errors at work, traffic accidents, as well as health and safety hazards (3, 4). Sleep plays an essential role in maintaining the body's normal functions. Even brief periods of insufficient sleep can induce alterations in cognitive functions such as attention and memory (5–7). Research has demonstrated that short-term sleep deprivation can impair various neurocognitive functions including alertness, executive attention, quick-wittedness, cognitive speed, and memory (8). Furthermore, the level of fatigue tends to escalate progressively with prolonged durations of sleep deprivation (9, 10). Therefore, establishing reliable and effective methods for monitoring and intervening in MF holds significant importance for specific occupational groups (11).

Current methods for monitoring MF primarily encompass both subjective and objective approaches. Subjective assessments are predominantly conducted using scales such as the Karolinska Sleepiness Scale (KSS) (12–14) and the Stanford Sleepiness Scale (SSS) (15, 16). These scales offer advantages in terms of simplicity, cost-effectiveness, and credibility (17). However, subjective assessments are susceptible to various influencing factors, resulting in a significant degree of subjectivity. Consequently, researchers have progressively developed objective assessment methods grounded in physiological and biochemical indicators. Heart rate variability (HRV) refers to the subtle fluctuations between successive heartbeats, specifically concerning changes in the RR interval. The magnitude of HRV is associated with autonomic nerve activity and serves to describe alterations in heart rate (HR) pulsation induced by sympathetic nervous system (SNS) and parasympathetic nervous system (PNS) activities within the autonomic nervous system. HRV can be derived from electrocardiogram (ECG) data (18, 19). In recent years, HRV has gained widespread application in studies focused on mental workload and fatigue assessment. Both time-domain and frequency-domain parameters exhibit characteristic variations under different MF conditions; thus, they are believed to possess potential for distinguishing levels of workload and evaluating degrees of MF. This capability has rendered HRV a prominent research focus within the domain of fatigue monitoring (20–23).

To mitigate the MF induced by sleep deprivation and enhance cognitive functions, central stimulant drugs represent one of the effective strategies (24, 25). Caffeine, a xanthine alkaloid compound, is known for its ability to temporarily alleviate drowsiness and restore mental clarity. Clinically, it is utilized for resuscitating individuals in a coma and as an adjunctive treatment for neurasthenia (26). Furthermore, caffeine is recognized as the most widely consumed central nervous system stimulant globally (27). The bioavailability of caffeine is notably high; it can traverse the blood-brain barrier with concentrations in the brain rising within just 5 min following oral administration. Peak concentrations in both plasma and brain are typically reached within 1 h. The metabolic half-life of caffeine in plasma ranges from approximately 2.5 to 4.5 h (28). For healthy adults, consumption levels up to 400 mg per day (equivalent to 5.5 mg/kg) do not present any health risks (29). Caffeine exerts moderate stimulation on the nervous system, alleviates fatigue, enhances mental flexibility, and increases responsiveness and alertness in complex environments (30). Research has demonstrated that acute intake of caffeine can significantly improve various cognitive and emotional parameters such as reaction time, attention span, and MF index (31). Crawford et al. (32) conducted a systematic study that further corroborated the efficacy of caffeine in enhancing attention and alertness among sleep-deprived individuals—findings consistent with those reported by the American Institute of Medicine.

In our study, we employed an open-label, non-randomized single-sequence crossover trial design combined with multidimensional subjective fatigue scales, cognitive ability assessments, and HRV monitoring to comprehensively evaluate the effects of low-dose caffeine oral tablets (COT) on alleviating MF.

## Materials and methods

### Materials

The COT was purchased from Changle Iron Kai Biotechnology Co., Ltd. in Changsha, China (Food Production License Number: SC10642011801427; Product Standard Number: GB24154; Specification: 3 g/piece). Each COT contained approximately 96 mg of caffeine as determined by HPLC (see Results), and participants in the experimental phase received a single tablet at 13:00. Magnesium Oxide was obtained from the China National Pharmaceutical Group (Batch No. 20230823). Trichloroacetic Acid was also sourced from the China National Pharmaceutical Group (Batch No. 20230614). Methanol of chromatographic grade was supplied by Dima Company (Batch No. R142695). The caffeine standard sample (C_8_H_10_N_4_O_2_, Purity ≥99%) was procured from CHEMService (Batch No. 12223900). The experimental water met the GB/T6682 Grade 1 standard. Instrumentation included HPLC (Thermo U3000), a balance (Mettler Toledo ME204E), a water bath (Shanghai Yiheng DKZ-2), and an ultrasonic cleaner (Xiaomei XM-300UVF). A 0.22 μm microporous aqueous filter membrane was utilized for filtration purposes, sourced from Ansep Experiment (86200873). Dynamic electrocardiography was conducted using an American Dym DMS 300-4A Holter monitor.

### Caffeine content determination

To verify the actual caffeine content, we followed GB 5009.139-2014 (33). The preparation of caffeine standard solutions is as follows: Caffeine Standard Stock Solution (2.0 mg/ml): Accurately weigh 20 mg of caffeine standard substance and transfer it into a 10 ml volumetric flask. Dissolve the substance in methanol and bring the solution up to the mark with additional methanol. Caffeine Standard Intermediate Solution (200 μg/ml): Transfer precisely 5.0 ml of the caffeine standard stock solution into a 50 ml volumetric flask, then dilute with water to the mark. Caffeine Standard Curve Working Solutions: From the intermediate solution, take aliquots of 0.1 ml, 0.5 ml, 1.0 ml, 2.5 ml, 5.0 ml, and 10.0 ml respectively into separate 100 ml volumetric flasks and dilute each with water to volume. This procedure yields standard solutions at concentrations of 0.2 μg/ml, 1 μg/ml, 2 μg/ml, 5 μg/ml, 10 μg/ml, and 20 μg/ml. It is recommended that these solutions be prepared fresh for immediate use.

Using the boiling water extraction method, precisely weigh 1.0300 g and 1.0176 g of COT (after passing through a 30-mesh sieve) and place them in a 250 ml conical flask. Add 200 ml of water and conduct a boiling water bath for 30 min, ensuring to shake the mixture during this process. Afterward, cool it for 1 min using running water, then add 5 g of magnesium oxide. Conduct an additional boiling water bath for 20 min before allowing the solution to cool to room temperature. Transfer the resulting mixture into a 250 ml volumetric flask and dilute with water up to the mark. Allow it to stand, then take the supernatant and filter it through a 0.22 μm filter membrane. Dilute this filtered solution tenfold, which will serve as the test sample solution.

### Nap deprivation

Ten healthy volunteers, aged between 22 and 45 years (4 males and 6 females), were selected for this study. The average age of the participants was 33.70 ± 7.06 years, with an average height of 163.20 ± 9.97 cm and a weight of 58.00 ± 10.02 kg. All volunteers exhibited a regular circadian rhythm and had a habitual practice of taking naps. They possessed at least a college degree, were right-handed, in good health, and did not engage in smoking, drinking alcohol, or consuming coffee or tea. One week prior to the experiment, participants maintained regular sleep patterns (≥7 h per day) and refrained from consuming any substances that could stimulate or inhibit the central nervous system; they also avoided high-intensity exercise as well as emotional fluctuations. The experiment was conducted in a controlled laboratory environment with constant temperature, humidity, and lighting conditions. Volunteers awoke at 08:00 and entered the testing room where they remained seated or standing until their departure at 20:00; during this period, they were prohibited from lying down or engaging in vigorous physical activity or high-stress tasks. This study received support from the hospital (991YJ-202304) along with approval from the ethics committee; all volunteers provided informed consent prior to participation.

**Figure 1:**
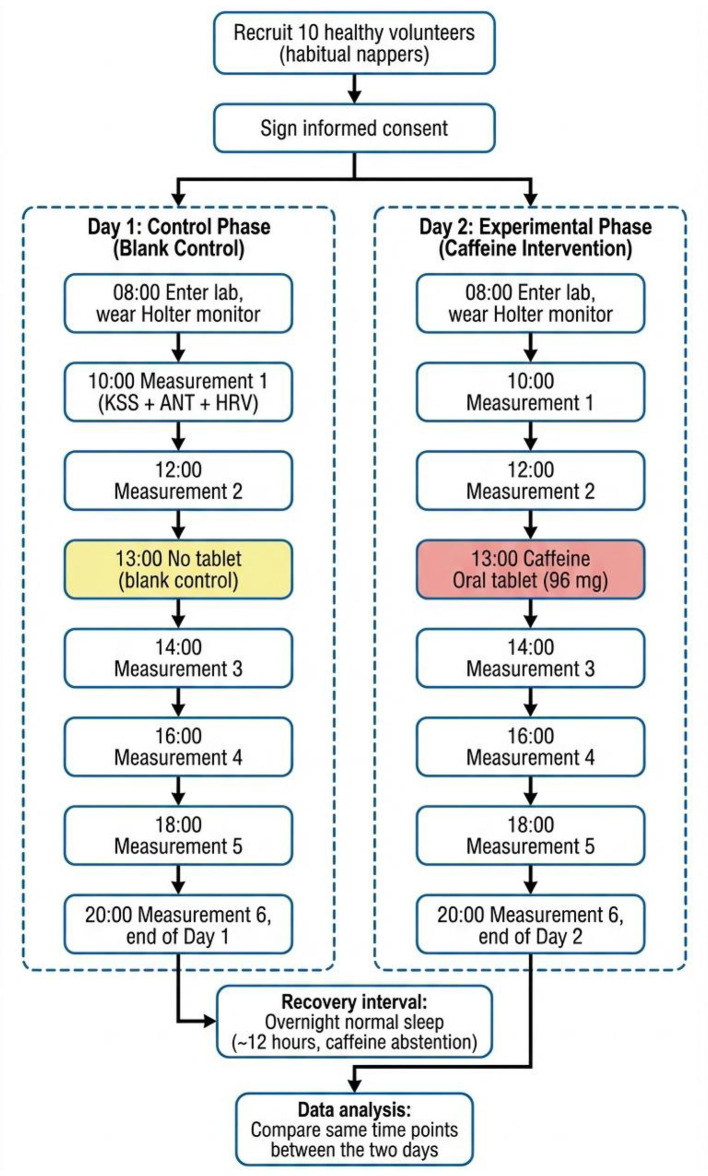
Study flow diagram. KSS, Karolinska sleepiness scale; ANT, attention network task; HRV, heart rate variability; COT, caffeine oral tablet.

Participants were required to wear Holter monitors before 08:00. Initially, they completed the Karolinska Sleepiness Scale (KSS) according to a predetermined schedule before commencing the Attention Network Test (ANT). This entire process lasted approximately 5 min. Data collection occurred synchronously every 2 h at designated time points: 10:00, 12:00, 14:00, 16:00, 18:00, and finally at 20:00. The data collected at 10:00 (2-h deprivation) served as baseline measurements. The study employed an open-label, single-sequence crossover design. On day 1 (control phase), participants underwent nap deprivation from 08:00 to 20:00 with no tablet administered at 13:00 (blank control). On day 2 (experimental phase), the same protocol was repeated, but each participant received one COT containing 96 mg of caffeine at 13:00. The two phases were separated by a 12-h overnight recovery interval (normal sleep from approximately 20:00 to 08:00), during which participants abstained from caffeine. Because the control phase involved no intervention rather than an active drug, a formal washout period was not required; the overnight recovery allowed return to baseline physiological status. The timing of intervention (13:00) was chosen to occur between the 12:00 and 14:00 measurements, allowing assessment of acute effects. A flow diagram of the study protocol is provided in Figure 1.

### KSS

The scale ranges from 1 to 9, where: 1 = extremely alert; 2 = very alert; 3 = alert; 4 = moderately alert; 5 = somewhat sleepy but not drowsy; 6 = exhibiting some tendency toward sleepiness; 7 = feeling sleepy but requiring minimal effort to remain awake; 8 = feeling sleepy and necessitating some effort to stay awake; 9 = extremely sleepy and demanding significant effort to maintain wakefulness.

### ANT

An effective method was employed to measure the response time (RT) and correct response rate (CR) of the participants. During the experiment, volunteers responded according to the directional arrows displayed on the screen. The left arrow (“←”) corresponded to the “I” key on the keyboard, while the right arrow (“ → ”) corresponded to the “E” key. Upon completion of the test, both RT and CR were recorded for analysis.

### HRV

The volunteers were equipped with a 24-h dynamic electrocardiogram (Holter) to record the electrocardiographic waveforms and analyze variations in HRV. HRV analysis was performed using the DMS Holter system's built-in software (DMS 300-4A, USA). The analysis window was set to 5-min segments at each time point. All participants remained seated in an upright position during ECG recording, with respiration unrestricted. Artifacts were manually corrected prior to analysis. The following indicators were assessed:

Time-domain indicators: HR: the average heart rate over the sampling period. SDNN: the standard deviation of RR intervals, which reflects the dispersion of these intervals. rMSSD: the root mean square of successive differences between adjacent RR intervals, providing insight into the shape of the histogram representing RR interval differences and components contributing to changes in HRV. pNN50: the percentage of consecutive RR interval differences exceeding 50 ms among all intervals, indicating heart rate fluctuations and parasympathetic nerve activity's influence on HRV.

Frequency-domain indicators include total power (TP), low frequency (LF), high frequency (HF), normalized low frequency (LFnorm or LFn), normalized high frequency (HFnorm or HFn), as well as LF/HF ratio, among others.

HRV, as an objective and real-time physiological index, offers distinct advantages in the assessment of fatigue (34). It serves as a reflection of the balance between SNS and PNS (35). These two systems exhibit antagonistic properties in their functions. Typically, during a resting state, PNS regulation predominates, maintaining the body's physiological equilibrium. Conversely, when an individual experiences fatigue, struggle, fear, tension, or excitement, SNS regulation becomes dominant and actively facilitates the body's response to crises. The time-domain, frequency-domain, and non-linear indices of HRV are influenced by various factors including age, gender, physical activity levels, sleep quality, and changes in body position (36, 37). A consensus among researchers suggests that the time-domain index SDNN reflects overall SNS and PNS activity (38); rMSSD indicates the regulatory influence of the PNS on heart rate (HR), while pNN50 is recognized as a significant marker for assessing neural activity within the PNS (39). In terms of frequency-domain analysis: TP represents total power of HRV and captures comprehensive variations in HRV; LF signifies SNS activity within cardiac function (40); HF denotes PNS activity related to heart function (41); finally, LF/HF ratio is commonly interpreted as an indicator reflecting the balance between SNS and PNS or specifically SNS regulation (42).

### Statistical analysis

Statistical analysis was performed using GraphPad Prism software. The data were presented as mean ± standard deviation. Comparisons were performed using one-way repeated measures ANOVA followed by Dunnett's test. *P*-value < 0.05 was considered statistically significant. Given the multiple HRV comparisons, the risk of type I error is acknowledged; results should be interpreted as exploratory.

## Results

### Caffeine content

#### Methodological validation

**Figure 2:**
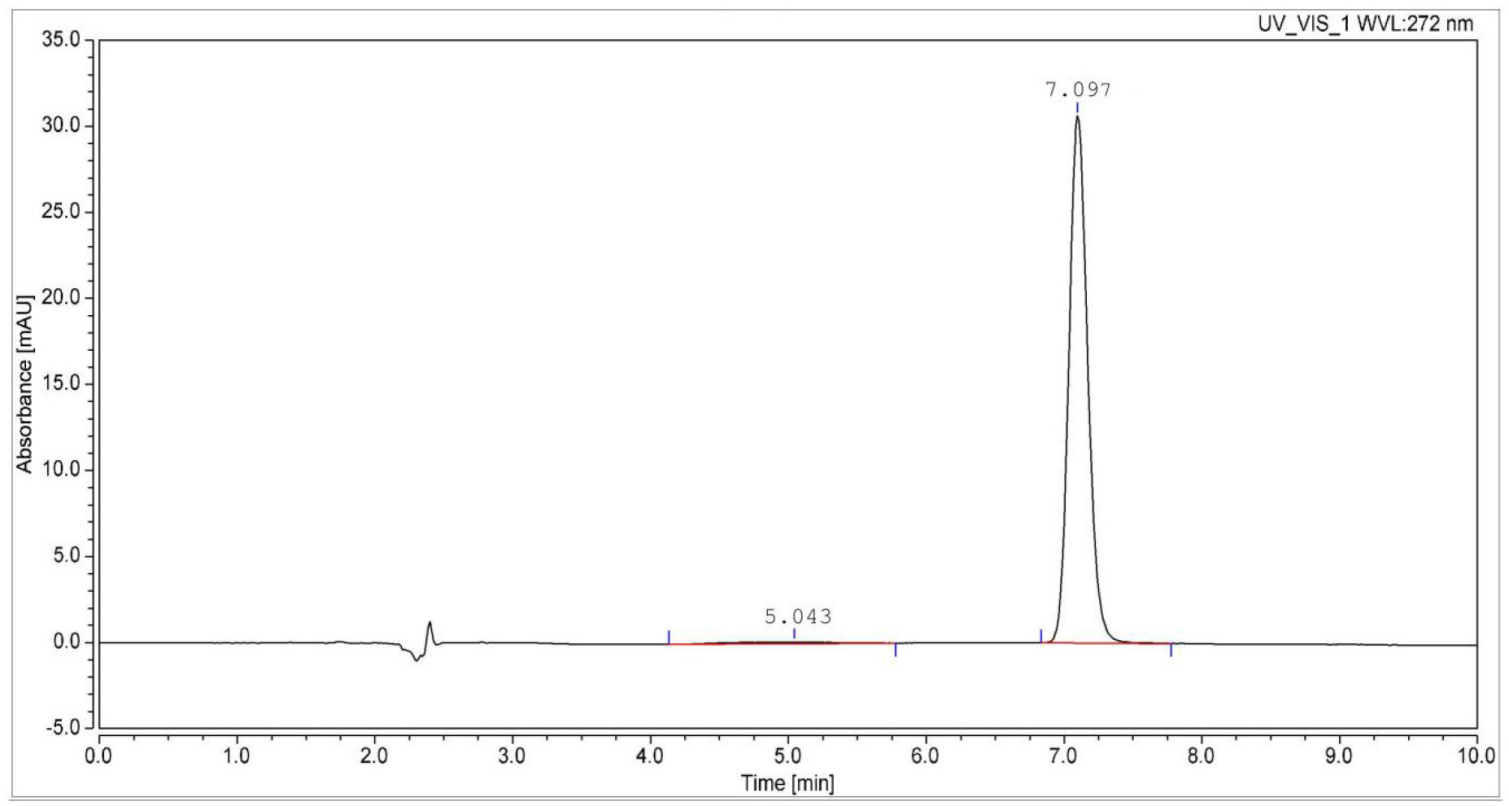
Chromatogram of caffeine standard sample.

**Figure 3:**
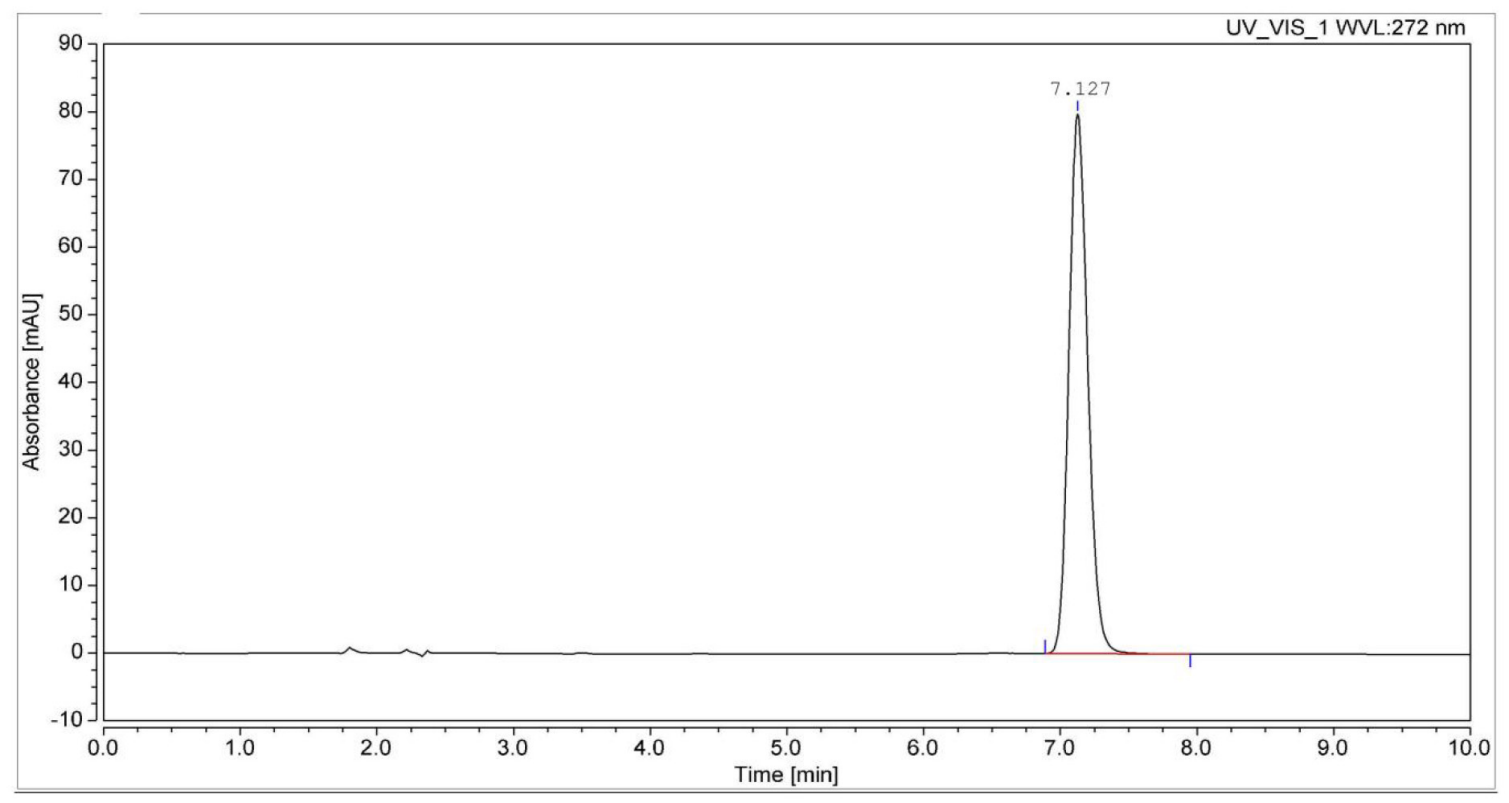
Chromatogram of COT.

According to GB 5009.139-2014, the chromatographic conditions employed were as follows: a C18 column (5 μm, 250 mm × 4.6 mm) was utilized with a column temperature set at 25 °C and an injection volume of 20 μl. The mobile phase consisted of methanol and water in a ratio of 30:70 (v/v), with a flow rate maintained at 1.0 ml/min; detection was performed at a wavelength of 272 nm. The results indicate that caffeine is effectively separated from impurities, with an analysis duration of 10 min (Figures 2, 3).

#### Standard curve

A standard curve was constructed by plotting concentration on the *x*-axis and peak area on the *y*-axis. The analysis demonstrated a strong linear relationship within the caffeine concentration range of 0.2–20 μg/ml. The regression equation obtained is *A* = 0.9639*C* – 0.0143 (*r* = 1, *n* = 6; Table 1).

**Table 1:** Data of the caffeine standard curve.

No	*C* (mg/L)	*A* (mAU·min)	Height (mAU)
1	0.2000	0.1925	1.215
2	1.0000	0.9473	6.054
3	2.0000	1.9267	12.307
4	5.0000	4.7464	30.474
5	10.0000	9.6663	62.048
6	20.0000	19.2569	125.060

#### Determination of caffeine content in COT

The caffeine content measured in the two parallel samples was 31.724 mg/g and 32.303 mg/g, respectively. The average concentration was calculated to be 32.0135 mg/g, indicating that each tablet contains approximately 96.0405 mg of caffeine (Table 2).

**Table 2:** Determination of caffeine content in COT.

No	Sample quantity (g)	A (mAU·min)	Total volume of the sample (L)	Caffeine content (mg/kg)
1	1.0300g	12.585	2.5	31,724
2	1.0176g	12.660	2.5	32,303

#### Changes in KSS of the volunteers

**Figure 4:**
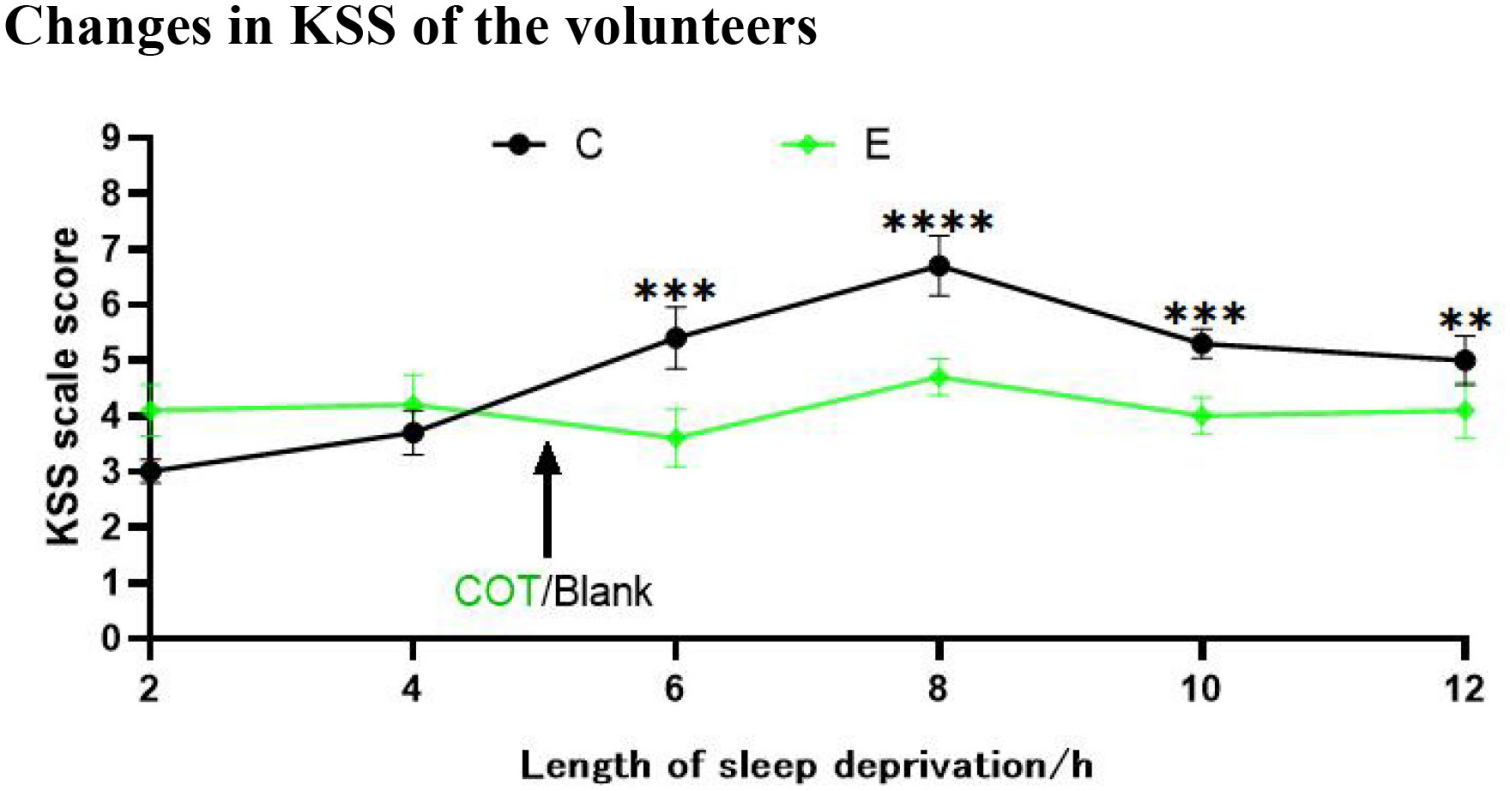
Changes in KSS scores of volunteers before and after COT intervention. Compared to the 2h, there was a significant difference (**P < 0.01, ***P < 0.001, ****P < 0.0001), Repeated-Measures ANOVA. Data are presented as mean ± SEM (*n* = 10 for each data point). C: control group; E: experimental group.

As the duration of sleep deprivation increases, the KSS score initially rises and then slightly decreases, peaking at 8 h of deprivation. When compared to baseline measurements taken after 2 h of deprivation, the control group exhibited a significant increase in KSS scores following 6–12 h of sleep deprivation (^**^P < 0.01). In contrast, within the experimental group that received COT treatment, no significant increase in KSS scores was observed (Figure 4).

#### Changes in ANT of the volunteers

**Figure 5:**
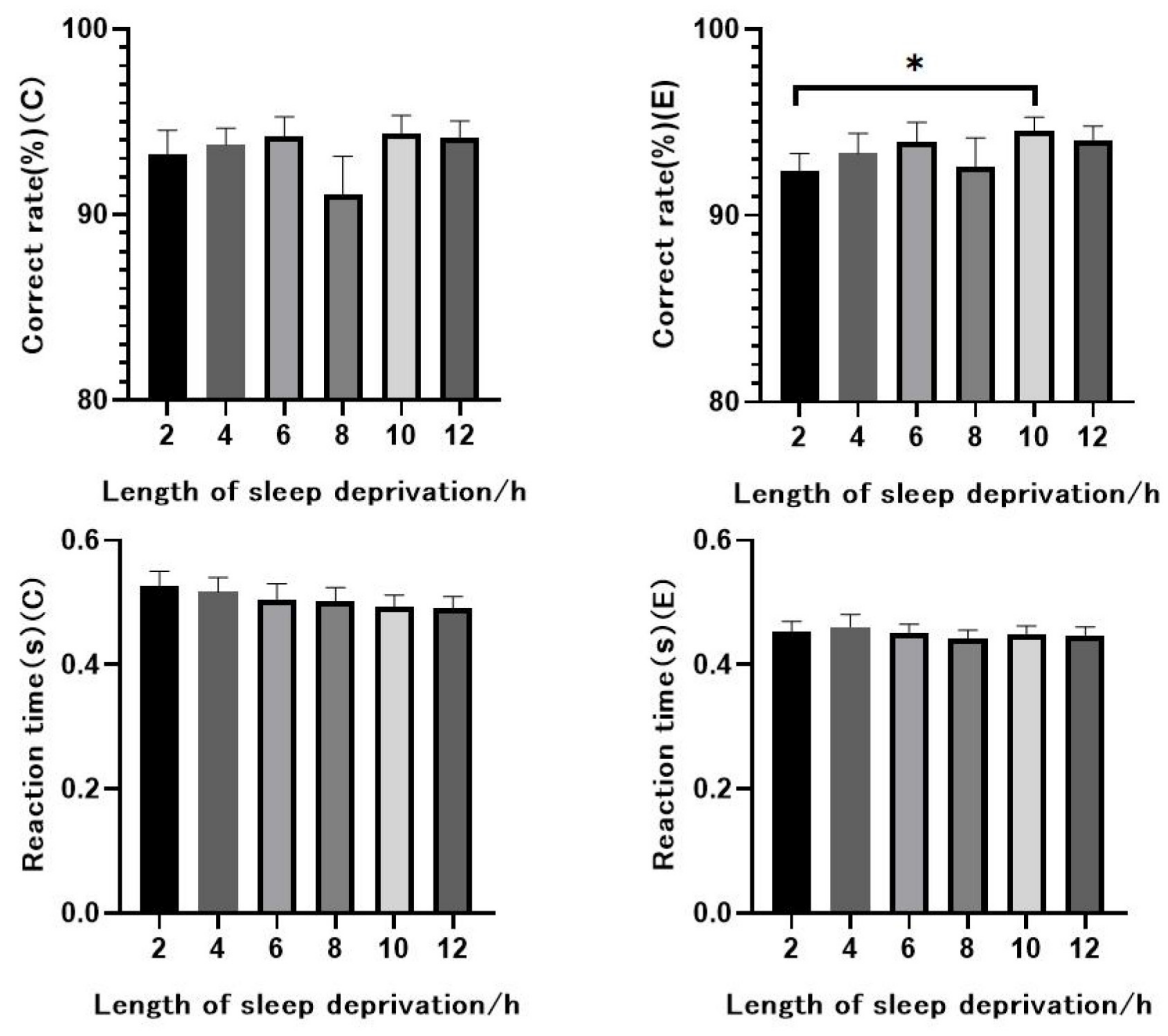
Changes in ANT levels of volunteers before and after COT intervention. Compared to the 2h, there was a significant difference (*P < 0.05), Repeated-Measures ANOVA. Data are presented as mean ± SEM (*n* = 10 for each data point). C: control group; E: experimental group.

In the ANT test, as the duration of deprivation increased, CR exhibited a pattern of initially decreasing followed by an increase, while RT progressively shortened. No significant changes were observed in CR and RT within the control group. Conversely, in the experimental group, CR was significantly elevated compared to baseline after 10 h of deprivation (*P* < 0.05; Figure 5). However, the control group did not show a statistically significant decline in ANT performance over time, which limits the strength of evidence for successful induction of measurable cognitive impairment by nap deprivation alone. This limitation should be considered when interpreting the caffeine-related improvements.

#### Changes in HRV of the volunteers

**Figure 6:**
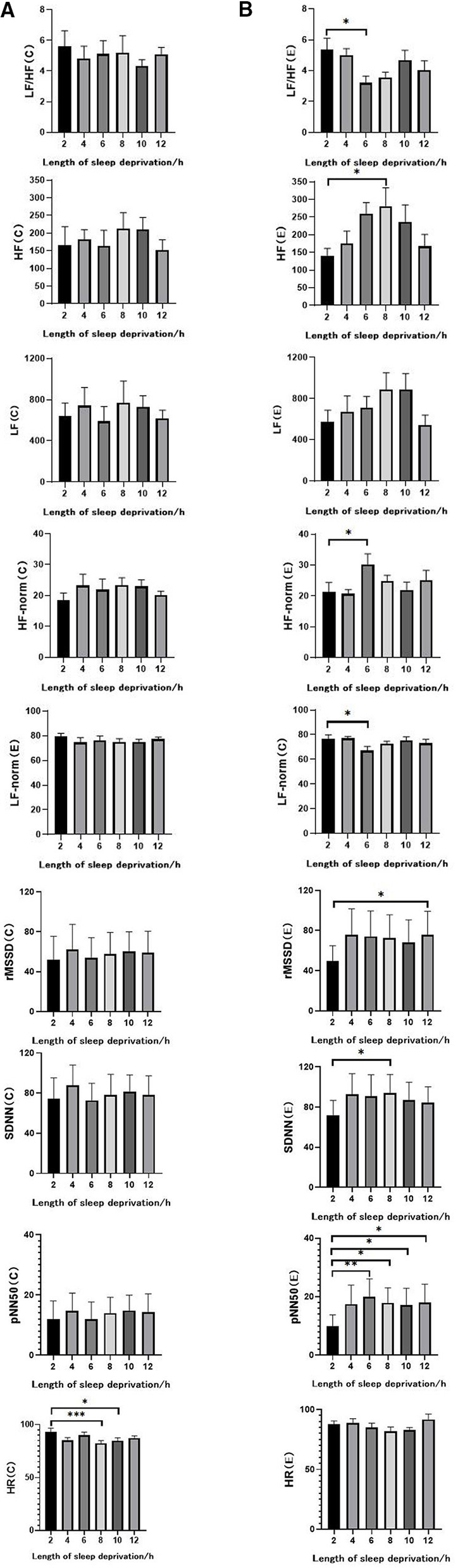
**(A)** Changes in HRV of frequency-domain indicators before and after COT intervention. Compared to the 2h, there were significant differences (*P < 0.05), with Repeated-Measures ANOVA. The data are presented as mean ± SEM (each data point *n* = 10). C: control group; E: experimental group. **(B)** Changes in HRV of time-domain indicators before and after COT intervention. Compared with 2h, there were significant differences (*P < 0.05, **P < 0.01, ***P < 0.001), with Repeated-Measures ANOVA. The data are presented as mean ± SEM (each data point *n* = 10). C: control group; E: experimental group.

Given the small sample size and the number of HRV indices examined, the following HRV findings should be considered exploratory. As the duration of sleep deprivation increases, the time-domain indices SDNN, pNN50, and rMSSD exhibit an upward trend, whereas HR shows a decline. In contrast, the frequency-domain indices LF and LFn decrease alongside fluctuations in LF/HF; conversely, fluctuations in HF and HFn increase. Compared to baseline measurements at 2 h (93.64 ± 3.63) of sleep deprivation, HR in the control group significantly decreased at both 8 h (82.26 ± 2.64) and 10 h (84.84 ± 2.89) of sleep deprivation (^*^P < 0.05), while no significant changes were observed in other indices. In the experimental group, rMSSD, SDNN, and pNN50 demonstrated significant increases at 12 h (49.50 ± 15.30 vs. 75.77 ± 23.49), 8 h (71.64 ± 14.95 vs. 93.76 ± 18.78), and during the period from 6 (10.01 ± 3.77 vs. 20.01 ± 6.14) to 12 h of sleep deprivation (^*^P < 0.05). Additionally, the frequency-domain indices LF/HF (5.37 ± 0.74 vs. 3.20 ± 0.44) and LFn (76.48 ± 3.47 vs. 67.12 ± 3.50) showed significant decreases after 6 h of deprivation (^*^P < 0.05), while HF (140.68 ± 20.46 vs. 281.40 ± 51.94) and HFn (21.20 ± 3.15 vs. 30.22 ± 3.44) exhibited significant increases at both 8 h and after 6 h of deprivation (^*^P < 0.05; Figure 6).

## Discussion

Our study employed an open-label, single-sequence crossover design based on the following considerations: (1) the primary objective of our investigation was to preliminarily assess the anti-fatigue effects of low-dose COT. Utilizing a self-preceding control effectively mitigates individual differences. (2) To prevent interference from residual caffeine effects, such as adaptive changes in adenosine receptors (43–48), the single-sequence design ensures stability in baseline conditions prior to subsequent interventions. (3) This design simplifies the process, reduces dropout risk, and allows all participants to potentially benefit from the intervention, aligning with ethical standards.

Sleep deprivation is a prevalent contributor to MF. While previous studies have primarily concentrated on complete sleep deprivation (49, 50), recent advancements in modern society have led many individuals to forgo their physiological sleep needs in favor of work or social activities. This trend has resulted in increasingly severe daytime sleepiness. Consequently, implementing short naps during the day has emerged as one of the most common strategies to mitigate fatigue and enhance performance (51–53). This study investigates the effects of nap deprivation on MF and reveals that both subjective assessments and cognitive tests indicate a progressive deterioration in fatigue levels as the duration of deprivation increases. Cognitive abilities decline significantly, peaking at 6–8 h of nap deprivation before partially recovering; however, these levels remain elevated compared to baseline measurements. These findings suggest that nap deprivation can effectively induce MF.

The following discussion interprets the HRV changes observed in this study in light of the above physiological principles. This study demonstrates that following nap deprivation, the heart rate (HR) in the control group exhibited significant changes, indicative of increased human fatigue and its impact on cardiac activity. The time-domain indices of rMSSD, SDNN, and pNN50 displayed fluctuating increases, while the frequency-domain indices of LF, LFn, and LF/HF showed decreased fluctuations alongside increased fluctuations in HF and HFn. These findings suggest a strengthening of PNS regulation coupled with a reduction in SNS regulation. This indicates that during the passive fatigue state induced by sleep deprivation, as fatigue intensifies, vagal tone gradually assumes dominance; consequently, physiological inhibitory mechanisms lead to a decrease in SNS tension. Moreover, these results imply that after sleep deprivation, there is an elevation in the body's regulatory capacity which mobilizes compensatory responses to counteract fatigue. Following caffeine intake (COT), multiple HRV parameters demonstrated significant alterations that further reinforced this compensatory effect. This suggests that caffeine may alleviate fatigue through modulation of autonomic nerve function. Previous studies have indicated that activation of the PNS branch—part of the relaxation system—occurs when MF increases along with HR elevation. The current experiment provides additional evidence supporting caffeine's substantial enhancement of this effect (54–56).

In addition to subjective and HRV findings, the ANT performance showed a significant improvement in correct response rate after caffeine intervention, even after 10 h of nap deprivation. This suggests that low-dose caffeine may exert a protective or enhancing effect on attentional processes under prolonged wakefulness. Previous studies have reported that caffeine can improve reaction time and accuracy in attention tasks following sleep loss (32, 57). Our results extend these observations to a nap deprivation model and a low-dose formulation. The preservation of attentional performance is particularly relevant for occupations requiring sustained attention, such as healthcare workers, pilots, and long-distance drivers. However, given the lack of a significant decline in the control group, this finding should be interpreted cautiously and replicated in future studies.

Caffeine, as an adenosine receptor antagonist, inhibits the binding of adenosine to its receptors in the brain, thereby eliciting sympathetic physiological responses such as wakefulness, alertness, and arousal (27, 58, 59). It also exerts extensive effects on both the cardiovascular and central nervous systems (60–62). This study observed that the duration of caffeine's beneficial effects was relatively short. Several factors may contribute to this observation: (1) the sample size was limited and individual differences (such as gender and female menstrual cycle) were present. (2) The administered caffeine dose was relatively low. Regulatory bodies including the FDA, EFSA, and Health Canada have indicated that for healthy adults, a daily intake of no more than 400 mg of caffeine is considered safe. In most cases, a minimum dose of approximately 200 mg per day is recommended for enhancing short-term performance (63, 64). Furthermore, individuals exhibit varying metabolic rates for caffeine due to genetic factors influenced by CYP1A2 and ADORA2A genes; thus a dosage of 96.0405 mg may be insufficient for some volunteers (65, 66). (3) Caffeine typically reaches peak effectiveness within 0.5–1 h post-consumption with effects lasting between 3 and 5 h; therefore it is possible that data collection did not encompass this peak effect window. (4) Fatigue resulting from sleep deprivation has multidimensional characteristics (67), while caffeine primarily alleviates cognitive fatigue (57, 68). (5) The cumulative nature of fatigue may partially obscure the intervention's efficacy; although comparisons made against baseline measurements taken 2 h earlier on the same day cannot entirely eliminate potential influences from learning or residual effects. Additionally, an insufficient sample size may diminish statistical power; hence future studies should aim to increase sample sizes in order to enhance the reliability of their conclusions.

Several limitations should be acknowledged. First, the small sample size (*n* = 10) and the absence of a placebo (only blank control) and randomization increase the risk of bias and limit the generalizability of the conclusions. Second, the open-label design may have introduced performance and detection bias. Third, the fixed sequence (control phase always before experimental phase) and repeated use of the ANT task may have led to learning effects, although baseline measurements were taken at the same time of day. Fourth, circadian rhythm was not fully controlled, as all measurements were conducted during daytime hours; changes in posture and respiration may also have influenced HRV. Fifth, only habitual nappers were included, which limits extrapolation to non-nappers. Sixth, sex differences and menstrual cycle effects in female participants were not analyzed due to the small sample. Seventh, only a single low dose of caffeine (96 mg) was tested, and the duration of its beneficial effect appeared short. Eighth, the lack of blinding and the multiple HRV comparisons increase the risk of type I error. Future studies with larger sample sizes, randomized double-blind placebo-controlled designs, and multiple caffeine doses are needed to confirm our preliminary findings.

## Conclusion

Future research could explore the use of non-invasive wearable HRV monitors for real-time fatigue monitoring (69–73), but this remains an investigational direction rather than a validated conclusion of the current study. In future research, the effects of varying doses of caffeine, gender differences, and circadian rhythms on HRV assessments of fatigue warrant further exploration. Additionally, integrating multi-dimensional indices such as electrocardiogram (ECG), cerebral blood oxygen levels, salivary cortisol concentrations, and saccadic eye movements with physiological indices and cognitive ability tests could provide a more comprehensive understanding. Through high-quality randomized double-blind controlled multicenter studies with large sample sizes, this assessment can be validated and correlated with other existing methods for detecting MF. Such efforts will provide a scientific basis for precise interventions targeting MF.

In summary, this pilot study provides preliminary evidence that low-dose caffeine is associated with favorable changes in subjective sleepiness, attentional performance, and HRV parameters in a nap deprivation model. The consistency across subjective, cognitive, and physiological measures supports the potential utility of HRV as an objective fatigue indicator. Nevertheless, due to the study's exploratory nature and limitations, these findings require confirmation in larger, randomized, double-blind, placebo-controlled trials before any firm conclusions can be drawn. In future research, the effects of varying doses of caffeine, gender differences, and circadian rhythms on HRV assessments of fatigue warrant further exploration. Additionally, integrating multi-dimensional indices such as electrocardiogram (ECG), cerebral blood oxygen levels, salivary cortisol concentrations, and saccadic eye movements with physiological indices and cognitive ability tests could provide a more comprehensive understanding (74). Through high-quality randomized double-blind controlled multicenter studies with large sample sizes, this assessment can be validated and correlated with other existing methods for detecting MF (75, 76). Such efforts will provide a scientific basis for precise interventions targeting MF.

## Data Availability

The original contributions presented in the study are included in the article/supplementary material, further inquiries can be directed to the corresponding authors.
